# Potential benefits of Rehmanniae Radix after ancient rice‐steaming process in promotion of antioxidant activity in rats' health

**DOI:** 10.1002/fsn3.3509

**Published:** 2023-06-22

**Authors:** Ying Zhang, Meng‐xi Wu, Hong‐mei Li, Jianhui Sun, Lu‐qi Huang, Yuan Yuan

**Affiliations:** ^1^ National Resource Center for Chinese Materia Medica China Academy of Chinese Medical Sciences Beijing China; ^2^ Institute of Chinese Materia Medica China Academy of Chinese Medical Sciences Beijing China; ^3^ State Key Laboratory of Dao‐di Herbs China Academy of Chinese Medical Sciences Beijing China

**Keywords:** antioxidant activity, catalpol, health‐promoting effects, hemoglobin genes, rehmannioside A, rice steam processed product of Rehmanniae Radix

## Abstract

Rice steam processed product of Rehmanniae Radix (RSRR), one of the processed products of Rehmanniae Radix (RR), is popular as an herbal medicine and food. However, the health‐promoting effects and mechanisms of RSRR are still unclear. In this study, 10‐week‐old Sprague–Dawley female rats were treated with different processed products of RR. No organ coefficient differences were observed between RSRR and the control group, indicating that RSRR did not cause damage to the rats. Compared with other RR products, superoxide dismutase, glutathione, and catalase levels were significantly higher and malondialdehyde levels were significantly lower in the RSRR group, indicating that RSRR exerted a better antioxidant effect. Gene expression analysis showed that hemoglobin genes (*Hba‐a1*, *Hba‐a2*, *Hbb‐bs*, *Hbb*, *Hbq1b*, *Hbb‐b1*, and *LOC103694857*) may be potential biomarkers to evaluate the antioxidant effect of RSRR. Antioxidation‐related signaling pathways in GO annotation, including cellular oxidant detoxification, hydrogen peroxide metabolic process, hemoglobin complex, and oxygen binding signaling pathways were significantly enriched, indicating these pathways may represent the antioxidant mechanism of RSRR. To explore the main active compounds primarily responsible for the antioxidant activity of RSRR, UPLC‐Q‐TOF‐MS was used and six components (catalpol, rehmannioside A, rehmannioside D, melittoside, ajugol, and verbascoside) were identified in rat serum. Catalpol and rehmannioside A were predicted to be the major active components by network pharmacology. These results suggested that RSRR exhibits antioxidant activity and has health‐promoting properties. This study provides a scientific basis for the antioxidant mechanism and clinical use of RSRR.

## Graphical abstract

Rice steam processed product of Rehmanniae Radix (RSRR), unearthed from the tomb of Haihunhou Liu He (emperor, 59 BC, Western Han Dynasty) in China, is a testament to the long history of steaming and ancient rice‐steaming method in China. RSRR showed health‐promoting effects by exerting antioxidant activity via enhancement of enzymatic and nonenzymatic systems and has health‐promoting properties. Antioxidation‐related signaling pathways may be the antioxidant mechanism. RNA‐seq analysis showed that hemoglobin genes can be used as biomarkers to evaluate the antioxidant effect. Catalpol and rehmannioside A may be the main active components to exert antioxidant effect of RSRR.

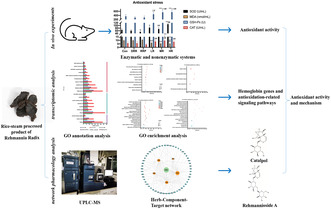

## 1 INTRODUCTION

Functional foods that contain a high proportion of effective constituents may be natural or processed products (Altun et al., 2021). Functional foods get increasing individual tendencies due to their effectiveness, nontoxicity, and capability of regulating body functions properties (Altun et al., 2021; Carneiro et al., 2020). Rehmanniae Radix (RR), considered a homology of medicine and food, was first recorded in *Shennong's Classic of Materia Medica* approximately 2000 years ago. RR is the tuber root of *Rehmannia glutionsa* Libosch and belongs to the Scrophulariaceae family. RR has anti‐inflammation and antioxidant properties, promoting diabetic wound healing and treatment of bone diseases (Bao et al., 2018). RR is widely used in Asian countries (Li et al., 2022).

Herbal drugs are processed to ensure safety or enhance efficacy according to theory and practice of traditional Chinese medicine (TCM; Yi et al., 2018; Zhang et al., 2019). Dried Rehmanniae Radix (DRR) and Rehmanniae Radix Praeparata (RRP), which are recorded in the Chinese Pharmacopeia (2020 edition), are two types of processed products of RR (Commission, 2020; Gong et al., 2019). There is a long history in China of using steaming and rice steam processing methods to prepare RSRR (Liu et al., 2017). Archeological research has confirmed that rice steam processed product of Rehmanniae Radix (RSRR), which was unearthed from the tomb of Haihunhou Liu He (59 BC, Western Han Dynasty) in Jiangxi Province in China, was processed by steaming, using rice as an auxiliary material (Zhu et al., 2021).

Rice is a food stable for over half of the world's population and has been used as an important adjuvant material in TCM processing for thousands of years (Meresa et al., 2020; Shikai & Peisong, 2021). The use of rice as a steaming auxiliary material for processing RR may be mainly related to preventing the interaction of ancient iron pots with RR (Zhu et al., 2021). However, the practice of steam processing of RR with rice has been lost over time. Meanwhile, as the earliest processed product of TCM, RSRR is not recorded in the Chinese pharmacopeia and is rarely studied. Whether rice as a processing auxiliary material of RR exerts further benefits merits further study. Meanwhile, it is unclear whether RR after ancient rice‐steaming method processing (RSRR), which was commonly used by the emperor Liu‐He of the Han Dynasty, has health benefits.

Oxidative stress is understood as an imbalanced condition between the proportion of oxidants and antioxidants (Luo et al., 2020). Oxidative stress contributes to the aging process, infertility, cancer, cardiovascular diseases, and other adverse human health consequences (Bisht et al., 2017; Li et al., 2019; Luo et al., 2020). The use of antioxidants to eliminate oxidants, maintain the biological redox steady states, and exert effects on diseases has been extensively studied (Pisoschi & Pop, 2015). Functional foods with essential constituents often exhibit antioxidative properties (Arooj et al., 2021) and it has been reported that RR has antioxidant activity (Li et al., 2022). However, current studies do not examine the antioxidant activity of RSRR. It is unclear whether RSRR as a functional food exerts antioxidant activity.

In this study, through in vivo experiments, network pharmacology analysis, and transcriptomic analysis, we aimed to elaborate on the health‐promoting effects and mechanism of RSRR. This is the first study to explore the antioxidant activity and mechanism of RSRR and to provide a basis for research and practical use in the future.

## 2 MATERIALS AND METHODS

### 2.1 Plant materials and preparation

Dried Rehmanniae Radix (DRR, lot number: 20201013) was collected from Henan Province, China. All samples were preserved in the State Key Laboratory of Dao‐Di Herbs, China Academy of Chinese Medical Sciences. RSRR was prepared as described previously (Wu et al., 2022): 200 g of roots were soaked in 40% water for 1 h, then 4 g of rice flour was added to atmospheric steam twice for 4 h each. RRP was prepared according to the method recorded in the Chinese Pharmacopeia (2020 edition) (Commission, 2020), and dried at 70°C to a moisture content of less than or equal to 15%.

The prepared DRR, RSRR, and RRP were extracted separately using the following methods: 100 g of the sample was soaked in 12‐time volume fresh water for 4 h, then extracted under reflux for 60 min; subsequently, the sample was soaked in 10‐time volume of water for the second time and extracted under reflux for 90 min. The combined extracts were concentrated under reduced pressure to a yield of 0.5 g mL^−1^.

### 2.2 Animal care and general protocol

Ten‐week‐old Sprague–Dawley female rats (160 ± 20 g) were purchased from Beijing Vital River Laboratory Animal Technology Co., Ltd. (License: SCXK (Jing) 2021‐0006). Rats were housed individually under environmentally controlled conditions (temperature 22 ± 2°C and humidity 50%–60%) with a light/dark cycle of 12 h, free access to water, and a standard laboratory diet. The study was performed in accordance with the guidelines approved by the Institutional Animal Care and Use Committee (IACUC) of the Institute of Chinese Materia Medica, CACMS (Approval number: 2021B223).

Animals used for in vivo experiments were treated for 30 days, whereas those used for transcriptomic analysis were treated for 4 days. Six groups (*n* = 5 in each group) were administered the following by gavage: (a–c) low‐, middle‐, and high‐dose RSRR solutions (LR, 3 g kg^−1^; MR, 6 g kg^−1^; HR, 12 g kg^−1^); (d) 6 g kg^−1^ of DRR solution; (e) 6 g kg^−1^ of RRP solution; and (f) an equal volume of water as control. Appropriate safety measures were taken for handling the animals during the treatment period. Doses were adjusted weekly according to the body weight of rats.

### 2.3 In vivo experiments

#### 2.3.1 Blood and organ collection

All rats were weighed and sacrificed at the end of the experiment. Blood samples from inferior vena cava were collected. After 20 min stand, blood samples were centrifuged at 2400 × *g* for 20 min to obtain serum samples, which were stored at −80°C for further use. Afterward, rat organ samples (kidney, liver, spleen, and thymus) were carefully removed, washed, and weighed. The organ coefficient was calculated for each organ using the formula: organ coefficient = organ mass/body weight × 100% (*n* = 5).

#### 2.3.2 Antioxidant capacity

Blood samples were centrifuged at 5500 × *g* for 20 min to extract serum for analysis. Superoxide dismutase (SOD), malondialdehyde (MDA), glutathione peroxidase (GSH‐Px), and catalase (CAT) levels were assayed using corresponding assay kits (Nanjing Jiancheng Bioengineering Institute, Nanjing, China) by ELISA method according to the protocols described previously (Ning et al., 2018). All measurements were performed in quintuplicate for every sample.

### 2.4 Transcriptome analysis

#### 2.4.1 RNA extraction

After treating for 30 days, rats were injected intraperitoneally with 10% chloral hydrate solution for anesthesia. Whole blood was collected from the inferior vena cava. Total RNA was extracted from the blood using TRIzol Reagent (Invitrogen) according to the manufacturer's instructions. Genomic DNA was removed using DNase I (TaKara). RNA quality was then determined by 2100 Bioanalyser (Agilent) and quantified using the ND‐2000 spectrophotometer (NanoDrop Technologies). Only high‐quality RNA samples (OD260/280 = 1.8–2.2, OD260/230 ≥2.0, RIN ≥6.5, 28S:18S ≥1.0, >1 μg) were used to construct sequencing library. Five biological duplications were performed in each group.

#### 2.4.2 RNA sequencing

cDNA library preparation was performed following the TruseqTM RNA sample preparation kit from Illumina according to the manufacturer's protocol. cDNA libraries were sequenced on an Illumina HiSeq X Ten/NovaSeq 6000 sequencer (2 × 150 bp read length) using standard protocols. Raw reads were trimmed and quality controlled by SeqPrep (https://github.com/jstjohn/SeqPrep) and Sickle (https://github.com/najoshi/sickle) with default parameters. The clean reads were then individually aligned to reference genome with orientation patterns using HISAT2 (http://ccb.jhu.edu/software/hisat2/index.shtml) software. Mapped reads of each sample were assembled by StringTie (http://ccb.jhu.edu/software/stringtie/).

#### 2.4.3 Differential expression analysis

Gene expression levels for each transcript were estimated using the transcripts per million (TPM) method. RSEM (http://deweylab.biostat.wisc.edu/rsem/) was used to quantify gene abundances. DESeq2/DEGseq/EdgeR was used to analyze differentially expressed genes (DEGs) with *Q*‐value ≤0.05, |log2FC| > 1, and *Q*‐value ≤0.05 (DESeq2 or EdgeR)/*Q*‐value ≤0.001 (DEGseq). In addition, GO and KEGG enrichment analyses were performed at Bonferroni‐corrected *p* ≤ .05.

### 2.5 Blood components analysis

#### 2.5.1 Serum sample preparation

Serum was processed via methanol precipitation. Each serum sample (600 μL) was added to 1.8 mL methanol and vortexed for 1 min. The pooled serum was then centrifuged at 13400 × *g* for 10 min at 4°C. The supernatant was transferred to a clean EP tube and evaporated until dry. The residue was dissolved in 200 μL of methanol, followed by apex mixing for 2 min and sonication for 10 min. Following centrifugation at 13800 × *g* for 15 min, the supernatant was filtered through a 0.22 μm membrane.

#### 2.5.2 UPLC–MS analysis of blood components

Blood components were analyzed by UPLC–MS (*n* = 5). Reference standards, including rehmannioside D, catalpol, verbascoside, melittoside, and ajugol, were purchased from Shanghai Yuanye Biotechnology Co., Ltd. Rehmannioside A was provided by Wuhan ChemFaces Co., Ltd. The purity of the standards was not less than 98%. Acetonitrile and formic acid (LC‐MS grade) were purchased from Thermo Fisher Scientific, Inc. Analytical grade methanol and acetonitrile were provided by Tianjin Dingshengxin Chemical Co., Ltd. Ultrapure water was prepared by Pacific‐T‐II System (Thermo).

Waters ACQUITY UPLC system coupled with a Q‐TOF SYNAPT G2‐Si high‐definition Mass Spectrometer (Waters Corporation) was used for blood component analysis through the ESI interface. Waters Empower II was used for data collection and data analysis. A Waters ACQUITY UPLC BEH C_18_ column (2.1 mm × 50 mm, 1.7 μm) was used for separation at a flow rate of 0.3 mL min^−1^ at 40°C. The injection volume was 2 μL. The mobile phase was 0.1% formic acid–water (A) and acetonitrile (B). The following gradients were used: 0–7 min, 2% → 22%B; 7–9 min, 22% → 25%B; 9–16 min, 25% → 44%B; 16–20 min, 44% → 55%B; 20–30 min, 55% → 99%B; 30–33 min, 99% → 50%; 33–35 min, 50% → 2%B; and 35–36 min, 2%B.

The UPLC system was connected to a triple‐quadrupole mass spectrometer (Waters Xevo TQ‐S) and operated in negative‐ion modes under the following conditions: capillary voltage of 3.0 kV and cone voltage of 35 V. The drying gas rate was set at 10 L min^−1^ at 350°C. The atomization pressure was set to 35 psi. The ion source temperature was 120°C. The full‐scan mode range was 50–1500 Da for acquiring the MS data.

#### 2.5.3 Network construction and core compounds analysis

Network pharmacology was used to predict the main active components. Based on the compounds detected in the blood, the predicted targets were collected from PharmMapper (http://www.lilab‐ecust.cn/pharmmapper), which selected normalized fit score >0.9. Relevant target gene names were obtained from UniProt (http://www.uniprot.org/uploadlists/). To obtain the core compounds exerting antioxidant effect, an herb compound–target interaction network was constructed using Cytoscape (Version 3.9.1).

### 2.6 Statistical analysis

Statistical analysis was performed using SPSS version 26.0 statistical software (IBM Corp.). Data are presented as mean ± standard deviation (*M* ± SD). Results were analyzed using one‐way ANOVA and *LSD* analysis. Differences were considered statistically significant at *p* < .05.

## 3 RESULTS

### 3.1 Antioxidant capacity

**FIGURE 1:**
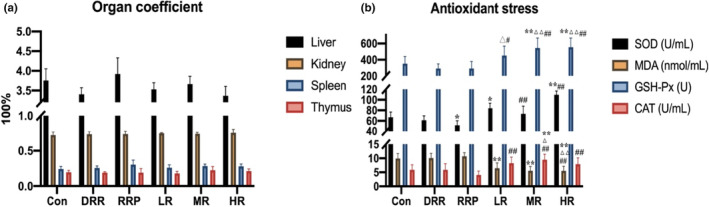
Function analysis of RSRR. (a) Organ coefficient; (b) Antioxidant stress. The values were presented as mean ± SD (*n* = 5). Significant differences with the Con group were designated as *p < .05 and **p < .01; with RR group designated as ^△^ *p* < .05 and ^△△^ *p* < .01; with RRP group designated as ^#^ *p* < .05 and ^##^ *p* < .01.

Organ coefficients are often used to represent the functional status of organs and provide a reference for drug safety evaluation (Yong et al., 2018; Zhang et al., 2014). There were no significant differences (*p* > .05) in the organ coefficient of rats treated with RR products (Figure 1a and Table 1), indicating that RR products treatment did not cause damage to rats.

**TABLE 1:** Organ coefficients (100%) in each group (*n* = 5).

	Liver	Kidney	Spleen	Thymus
Con	3.756 ± 0.296	0.722 ± 0.045	0.242 ± 0.034	0.195 ± 0.026
DRR	3.406 ± 0.168	0.734 ± 0.041	0.257 ± 0.028	0.191 ± 0.013
RRP	3.922 ± 0.410	0.736 ± 0.044	0.304 ± 0.063	0.191 ± 0.056
LR	3.529 ± 0.167	0.747 ± 0.008	0.261 ± 0.039	0.180 ± 0.029
MR	3.665 ± 0.196	0.741 ± 0.019	0.283 ± 0.028	0.225 ± 0.151
HR	3.364 ± 0.241	0.755 ± 0.054	0.280 ± 0.033	0.212 ± 0.031

The effects of treating different RR products on the antioxidant capacity of rats were compared. Compared with the control group, the SOD level was higher in RRP, LR, and HR groups (*p* < .01 or *p* < .05); GSH level was higher in MR and HR groups (*p* < .01); CAT level in MR group was also higher (*p* < .01), while the LR, MR and HR groups had a lower MDA level (*p* < .01). Compared with the DRR and RRP groups, the SOD, GSH, and CAT levels in RSRR group were significantly higher while MDA level was significantly lower (*p* < .01 or *p* < .05) (Figure 1b and Table 2). These results showed that RR after steam processing with rice exerted a strong antioxidative effect. In addition, RSRR showed stronger antioxidative activity than DRR and RRP.

**TABLE 2:** Antioxidant activity of RSRR (*n* = 5).

Group	SOD (U mL^−1^)	MDA (nmol mL^−1^)	GSH‐XP (U)	CAT (U mL^−1^)
Con	66.68 ± 10.05	9.93 ± 1.79	352.50 ± 87.65	5.90 ± 1.75
DRR	61.26 ± 8.25	10.06 ± 1.71	291.43 ± 56.77	5.90 ± 2.16
RRP	51.43 ± 8.74*	10.69 ± 1.27	291.43 ± 86.93	4.07 ± 1.36
LR	84.10 ± 9.27*	6.47 ± 1.88**	451.07 ± 117.09^△,#^	5.90 ± 1.75^##^
MR	73.43 ± 14.43^##^	5.57 ± 1.50**	546.43 ± 122.81**^,△△,##^	9.49 ± 1.75**^,△,##^
HR	109.67 ± 7.06**^,##^	5.51 ± 1.62**^,△△,##^	555.00 ± 114.06**^,△△,##^	7.86 ± 2.3^##^

*Note*: The values were presented as mean ± SD. Significant differences with the Con group were designated as *p < .05 and **p < .01; with DRR group were designated as ^△^ *p* < .05 and ^△△^ *p* < .01; with RRP group were designated as ^#^ *p* < .05 and ^##^ *p* < .01.

### 3.2 Gene expression in serum

**FIGURE 2:**
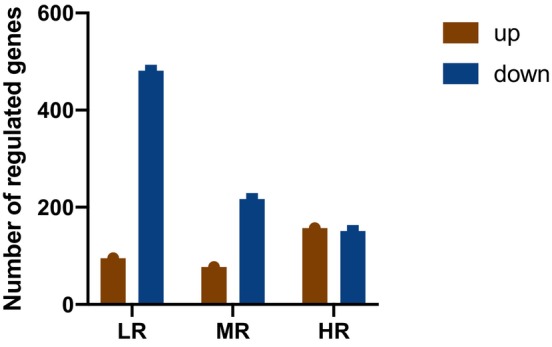
The number of regulated genes after different doses of treatment of RSRR. Brown color represents up‐regulated genes and blue color represents down‐regulated genes. All measurements were performed in quintuplicate for every sample.

**FIGURE 3:**
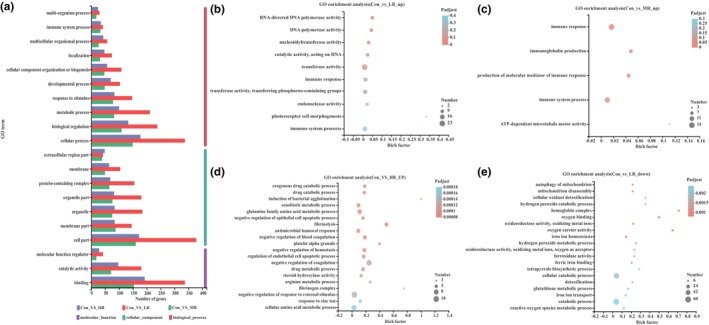
GO annotation and functional analysis of DEGs in each group. (a) GO annotation. The results are summarized in three main categories: molecular function, cellular component, and biological process. (b) GO enrichment analysis of upregulated DEGs in LR group. (c) GO enrichment analysis of upregulated DEGs in MR group. (d) GO enrichment analysis of upregulated DEGs in HR group. (e) GO enrichment analysis of downregulated DEGs in LR group.

Serum transcriptome analysis was conducted using RNA‐seq. A total of 406.99 Gb of clean data were generated, with clean reads aligned at an average alignment rate of 95.36%–96.76%, indicating that RNA‐seq data have relatively high performance. Compared with the control group, there were a total of 1178 DEGs in RSRR treated groups (Figure 2), of which 576 DEGs were found in LR group, including 95 upregulated and 481 down‐regulated genes; 294 DEGs in MR group, including 77 upregulated and 217 downregulated genes; and 308 DEGs in HR group, including 157 upregulated and 151 downregulated genes. Compared with the control group, GO annotation revealed that DEGs in LR, MR, and HR groups were primarily involved in response to stimulus; and were binding, catalytic activity, and molecular function regulator; and were localized to cell part, membrane part, organelle part, organelle, and protein‐containing complexes (Figure 3a). KEGG enrichment analysis is shown in Figure S1.

GO annotation also showed that upregulated genes in the LR group had RNA‐directed DNA polymerase activity, DNA polymerase activity, nucleotidyltransferase activity, and catalytic activity, acting on DNA (Figure 3b); and the upregulated genes in the MR group were primarily involved in immune response, the production of immunoglobulins, and molecular mediator of the immune response (Figure 3c). The upregulated genes in HR group were mainly involved in fibrinolysis, negative regulation of coagulation, negative regulation of hemostasis, regulation of blood coagulation, and fibrinogen complex (Figure 3d).

The downregulated genes in the LR group were mainly involved in the detoxification of cellular oxidants, hemoglobin complex, oxygen binding, hydrogen peroxide catabolism, oxidoreductase activity, oxidizing metal ions, oxygen carrier activity, iron‐ion homeostasis, ferric iron binding, and hydrogen peroxide metabolic process (Figure 3e). The top 4 oxidation‐related pathways were of great importance for the antioxidant effects of RSRR including cellular oxidant detoxification, hydrogen peroxide metabolic process, hemoglobin complex, and oxygen binding signaling. Compared with the control group, the transcription levels of *Hba‐a1*, *Hba‐a2*, *Hbb‐bs*, *Hbb*, *Hbq1b*, *Hbb‐b1*, and *LOC103694857* genes involved in these pathways were significantly decreased in RSRR group. It suggested that these hemoglobin genes could be considered potential biomarkers to evaluate the antioxidant effect of RSRR.

### 3.3 Identification of blood components in rat

**FIGURE 4:**
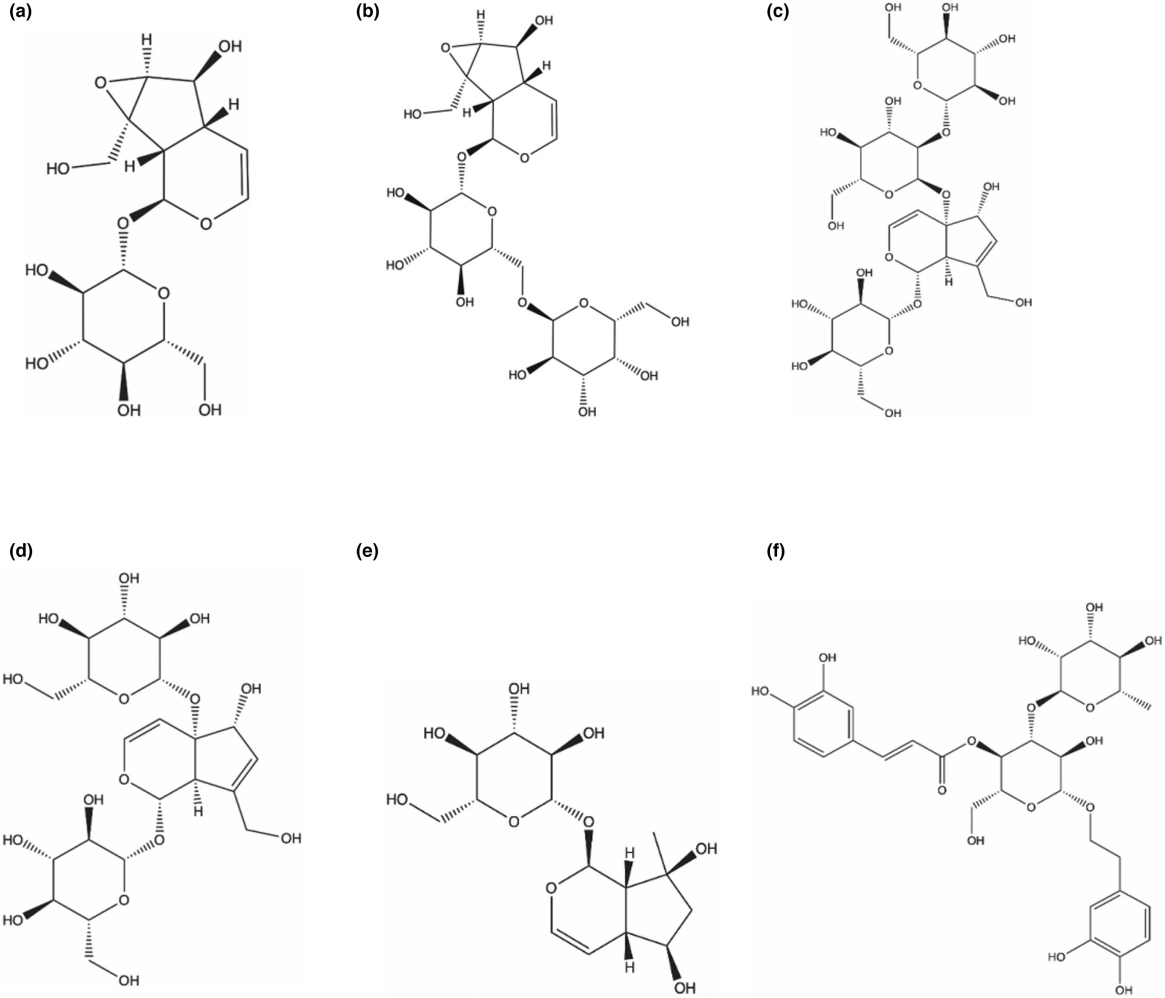
Structure of each compound. (a) Catalpol, (b) Rehmannioside A, (c) Rehmannioside D, (d) Melittoside, (e) Ajugol, (f) Verbascoside.

The total ion chromatography (TIC) of each serum group is shown in Figure S2. By comparing accurate m/z, fragment iron, and standards with mass error below 5 ppm, six prototype components belonging to iridoid glycosides and phenylpropane glycosides, including catalpol, rehmannioside A, rehmannioside D, melittoside, ajugol, and verbascoside, were detected in serum (Figure 4 and Table 3).

**TABLE 3:** Analysis results of prototype products in each administration group.

No.	*t*_R_ (min)	Identification	Molecular formula	[M − H] or [M + FA − H]	m/z	m/z of fragment iron	LR	MR	HR	DRR	RRP
1	1.87	Catalpol	C_15_H_22_O_10_	M + FA − H	407.1191	199.0611, 169.0546	+	+	+	+	+
2	2.37	Rehmannioside A	C_21_H_32_O_15_	M + FA − H	569.1747	323.0971, 199.0602	+	+	+	+	+
3	2.64	Rehmannioside D	C_27_H_42_O_20_	M + FA − H	731.2275	505.1549, 179.0541	+	+	+	+	+
4	2.75	Melittoside	C_21_H_32_O_15_	M + FA − H	569.1445	463.1469, 343.1021	+	+	+	+	+
5	3.29	Ajugol	C_15_H_24_O_9_	M − H	393.1399	329.1221, 167.0711	+	−	+	+	+
6	7.29	Verbascoside	C_29_H_36_O_15_	M − H	623.2020	461.1651, 161.0243	−	−	−	+	−

*Note*: “+” represents exist and “−” represents does not exist.

### 3.4 Screening of core functional compounds

**FIGURE 5:**
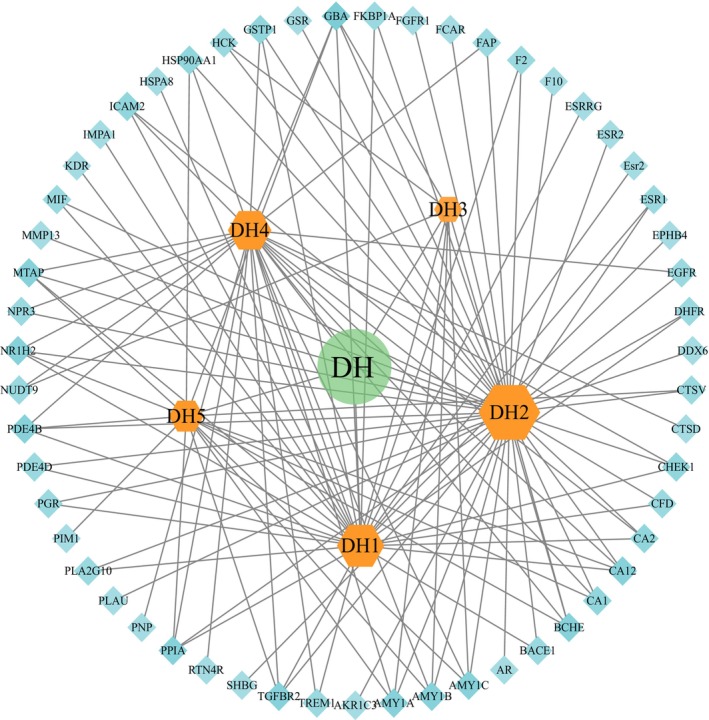
Herb component–target network. The gene targets are described as blue square. Orange hexagon stands for compounds. DH represents RSRR; DH1 represents Catalpol; DH2 represents Rehmannioside A; DH3 represents Rehmannioside D; DH4 represents Melittoside; DH5 represents Ajugol. Lines stand for the relationship between compounds and target nodes.

After normalizing gene names and eliminating overlaps by UniProt (Table S1), 55 target genes were selected for further analyses. An herb compound–target network was constructed (Figure 5), which included 61 nodes and 123 compound–target interactions. The degrees of nodes DH1 and DH2 were 29 and 44, respectively (Table 4), suggesting that compounds catalpol and rehmannioside A may be the key compounds for the function of RSRR.

**TABLE 4:** Basic information on the main active ingredients of RSRR.

	Compound	Degree value
DH1	Catalpol	29
DH2	Rehmannioside A	44
DH3	Rehmannioside D	9
DH4	Melittoside	25
DH5	Ajugol	16

## 4 DISCUSSION

As the earliest processed product of TCM, the rice steam processed product of Rehmanniae Radix (RSRR) unearthed from the tomb of Haihunhou Liu He (an Emperor, 59 BC) gets much attention in the world. The rice steam processing method was restored in our previous study (Wu et al., 2022), however, no pharmacological studies have been reported until now. To revive this ancient lost product of RSRR, the health‐promoting effects of RSRR which was used by the emperor were evaluated using network pharmacology analysis, transcriptomic analysis, and in vivo experiments.

Free radicals threaten human health and can lead to a series of chronic diseases (Hu et al., 2021). Improving antioxidant activity can scavenge free radicals and is a beneficial way to prevent disease (Chen & Huang, 2019). The antioxidant activity of Rehmanniae Radix has attracted much attention in recent years (Kim et al., 2017; Li et al., 2022; Liu et al., 2020). Catalpol, in particular, exerts strong antioxidant activity by enhancing endogenous antioxidant enzymatic activities and inhibiting free radical generation (You et al., 2021; Zhang et al., 2020). It is reported that catalpol can reverse abnormal levels of SOD, GSH, MDA, and CAT (Kim et al., 2009; You et al., 2021). SOD and CAT function as enzymatic systems that directly eliminate ROS (Xu et al., 2018). MDA is an indicator of lipid peroxidation. GSH is an endogenous antioxidant that catalyzes the reduction of hydrogen peroxide and other peroxides (Ning et al., 2018). The present study confirmed the antioxidant activity of RSRR, and SOD, GSH‐Px, and CAT levels were improved in rats after treating with RSRR, while MDA levels were significantly decreased, indicating that enzymatic and nonenzymatic systems were strengthened to prevent oxidative damage, suggesting that RSRR exerted a better antioxidative activity than DRR and RRP. This also implies that rice promotes antioxidant capacity as a processing aid for RR. The components with poor stability are easy to decompose during processing. Due to heating time and times difference, the degree of component decomposition varies, which leads to different compositions and contents among the processed products of TCM. A previous study showed that the quantity and quality of chemical components differ with or without rice, and with or without steam processing (Zhu et al., 2021), which may lead to different antioxidant capacities of different products of RR. TCM theory believes that adjuvant materials help to improve medicine efficacy (Zhang et al., 2019). Rice as an adjuvant material may be also contribute to the different antioxidant capacities of RR products. On the other hand, components absorbed into the blood are thought to exert health effects (Wang et al., 2021). In this study, the analysis of chemicals in the blood was carried out, however, chemical content differences were not analyzed. Varying chemical contents may also lead to varying antioxidant capacities.

Transcriptome analysis provides a detailed characterization of gene expression (Ding et al., 2020), providing the necessary link between the genetic information of the genome and the biological function of the proteome. RNA‐Seq was used in this study to obtain a global view of genetic changes in rats after RSRR feeding. GO enrichment analysis results showed that DEGs were closely associated with the four key pathways, including cellular oxidant detoxification, hydrogen peroxide metabolic process, hemoglobin complex, and oxygen‐binding signaling, which are related to the cellular redox state.

Hydrogen peroxide (H_2_O_2_), a by‐product of aerobic metabolism, can lead to oxidative damage (Giorgio et al., 2007). Reducing oxidative stress helps protect the body from an excess of reactive oxygen species (ROS). H_2_O_2_ can be converted or decomposed to H_2_O by enzymes such as SOD, GSH‐Px, and CAT (Armogida et al., 2012; Ye et al., 2008). Significant level changes in SOD, GSH‐Px, and CAT were observed in the in vivo study. This supported that the hydrogen peroxide metabolic process pathway might be essential for the antioxidant effect of RSRR.

Hemoglobin (Hb), which exhibits oxygen binding, can transport O_2_ from lungs to tissues (Nagatomo et al., 2017). Free Hb, which is detrimental to the human body, participates in diverse redox processes and may cause a cascade of lipid oxidation reactions (Mollan & Alayash, 2013). Hb consists of two pairs of identical globin chains, including the α‐like and β‐like chains. α‐Hemoglobin chains mediate the generation of reactive oxygen species and exacerbate oxidative damage. These reactive oxygen species interact with heme or heme‐derived iron to catalyze the production of hydroxyl radicals and reactive lipid radicals (Scott et al., 1993). The heme iron atoms are converted from the ferrous (Fe^2+^) to the ferric (Fe^3+^) form (Mollan & Alayash, 2013). β‐Hemoglobin chain synthesis is regulated by α‐chains (Wolf et al., 1973). The α‐ and β‐hemoglobin monomers exhibit high oxygen affinity (Richter et al., 2009). However, the unbalanced synthesis of α‐ and β‐chains, which is probably triggered by the precipitation of excess α‐chains, results in heme release and oxidative damage (Vallelian et al., 2015).

Excess ROS is extremely detrimental to organisms (Sharma et al., 2012). Apart from the transportation of O_2_ in the blood and enhancement of O_2_ supply to organs, Hb also serves as intracellular regulation of ROS homeostasis and detoxification of ROS (Burmester et al., 2018; Hieu et al., 2022). In this study, results showed that expression levels of *Hba‐a1*, *Hba‐a2*, *Hbb‐bs*, *Hbb*, *Hbq1b*, *Hbb‐b1*, and *LOC103694857* in rat serum were significantly downregulated after RSRR feeding. It suggested that RSRR might decrease hemoglobin through ROS signaling. We, therefore, speculated that RSRR may maintain oxygen homeostasis and protect against oxidative stress by decreasing the expression level of hemoglobin genes. Hemoglobin genes of *Hba‐a1*, *Hba‐a2*, *Hbb‐bs*, *Hbb*, *Hbq1b*, *Hbb‐b1*, and *LOC103694857* may serve as potential target genes to evaluate the antioxidant activity of RSRR.

Traditional Chinese medicine is characterized as multicomponent, multitarget, and multipathway. Serum pharmacochemistry believes that blood components are the active ingredients that play a therapeutic role (Wang et al., 2021). Five compounds of RSRR were identified in the serum using UPLC‐Q‐TOF‐MS. These compounds are iridoid and phenylpropanoid glycosides, and important components of RR that exert a wide range of pharmacological activities (Xue et al., 2022). Catalpol, ajugol, and rehmannioside A were reported to possess various biological properties, such as anti‐inflammation, antioxidant, and antiapoptosis (Jiang & Zhang, 2020; Sun et al., 2019; You et al., 2021; Zhang et al., 2021). Rehmannioside D possesses antiangiogenesis activity (Chen et al., 2020). Melittoside exerts antithrombotic activity (Gong et al., 2021). These five compounds were the basis for RSRR to exhibit antioxidant activity.

To determine the main active compounds primarily responsible for the antioxidant activity of RSRR, these five compounds were subsequently used in network construction. Based on the network pharmacological analysis, catalpol and rehmannioside A were considered as the main active compounds in RSRR due to the highest degree on the herb–compound–target network.

## 5 CONCLUSION

In this study, the health‐promoting effects and mechanisms of RSRR were analyzed. To our knowledge, this is the first study to systematically investigate the ancient rice steam processing method and antioxidant activity of RSRR. RSRR showed health‐promoting effects through exerting antioxidant activity by enhancing enzymatic and nonenzymatic systems. RSRR exerted a better antioxidative activity than DRR and RRP. Four key pathways (cellular oxidant detoxification, hydrogen peroxide metabolic process, hemoglobin complex, and oxygen binding signaling) and hemoglobin genes (*Hba‐a1*, *Hba‐a2*, *Hbb‐bs*, *Hbb*, *Hbq1b*, *Hbb‐b1*, and *LOC103694857*) were closely related to the antioxidant mechanism of RSRR. Six components including rehmannioside D, catalpol, melittoside, ajugol, verbascoside, and rehmannioside A were absorbed in rat serum. Catalpol and rehmannioside A are predicted as the main active components that exerted antioxidant effects. The above results showed the beneficial effects of RSRR, which is commonly used by the Emperor of the Han Dynasty. This is the first study to explore the health‐promoting effects and mechanisms of RSRR, which provides a scientific basis for future research and practical use.

## AUTHOR CONTRIBUTIONS

**Ying Zhang:** Investigation (equal); writing – original draft (lead). **Meng‐xi Wu:** Investigation (lead). **Hong‐mei Li:** Formal analysis (supporting); writing – review and editing (supporting). **Jianhui Sun:** Investigation (supporting). **Lu‐qi Huang:** Conceptualization (lead). **Yuan Yuan:** Conceptualization (lead).

## CONFLICT OF INTEREST STATEMENT

The authors declare that they have no conflicts of interest.

## Supporting information


Appendix S1

## Data Availability

The data that support the findings of this study are available on request from the corresponding author.
