# Supplementary material for: Potential benefits of Rehmanniae Radix after ancient rice‐steaming process in promotion of antioxidant activity in rats' health
Source: Food Sci Nutr. 2023 Jun 22;11(9):5532–42. doi: 10.1002/fsn3.3509 (PMC10494654; doi:10.1002/fsn3.3509)
Supplement: Supplementary file 1 — Appendix S1 [file FSN3-11-5532-s001.docx]

Supplementary Information

1. KEGG enrichment analysis of DEGs

For KEGG enrichment analysis in LR group compared with Con group (**Figure S1 A and Figure S1 B**), enriched metabolic pathways were ribosome, metabolic pathway, glutathione metabolism, arginine and proline metabolism, ubiquitin mediated proteolysis and so on. Up-regulated genes in MR group were enriched in glycine serine and threonine metabolism (**Figure S1 C**). The main enriched pathways in HR group were ribosome, tyrosine metabolism and pyrimidine metabolism and others (**Figure S1 D and Figure S1 E**).

There were no significant enrichment entries for differentially expressed genes between other groups and Con group.

**Figure S1.** The KEGG enrichment analysis of DEGs. A represents upregulated DEGs in LR group. B represents down regulated DEGs in LR group. C represents upregulated DEGs in MR group. D represents upregulated DEGs in HR group. E represents down regulated DEGs in HR group.

1. TIC of blood serum in each group of rats.

**Figure S2.** TIC of blood serum in each group of rats. A: TIC of LR group; B: TIC of MR group; C: TIC of HR group; D: TIC of DRR group; E: TIC of RRP group

1. Normalized target genes of each blood compound

**Table S1.** The corresponding target genes of each blood compound

| Compound | Gene | Compound | Gene | Compound | Gene | Compound | Gene |
| --- | --- | --- | --- | --- | --- | --- | --- |
| DH1 | PPIA | DH2 | AKR1C3 | DH2 | PGR | DH4 | BCHE |
| DH1 | CA2 | DH2 | HCK | DH2 | CHEK1 | DH4 | RTN4R |
| DH1 | CFD | DH2 | SHBG | DH2 | CA12 | DH4 | CHEK1 |
| DH1 | MIF | DH2 | F10 | DH2 | ICAM2 | DH4 | CA2 |
| DH1 | PDE4D | DH2 | BACE1 | DH2 | NPR3 | DH4 | PPIA |
| DH1 | NR1H2 | DH2 | F2 | DH2 | DDX6 | DH4 | PNP |
| DH1 | BACE1 | DH2 | GBA | DH2 | PLAU | DH4 | CA1 |
| DH1 | HSP90AA1 | DH2 | MIF | DH2 | NR1H2 | DH4 | TGFBR2 |
| DH1 | F2 | DH2 | GSTP1 | DH2 | FKBP1A | DH4 | NUDT9 |
| DH1 | TGFBR2 | DH2 | CFD | DH2 | FCAR | DH4 | AMY1A |
| DH1 | GSTP1 | DH2 | ESR1 | DH2 | EGFR | DH4 | AMY1B |
| DH1 | ESR1 | DH2 | DHFR | DH3 | BCHE | DH4 | AMY1C |
| DH1 | HSPA8 | DH2 | MTAP | DH3 | NUDT9 | DH4 | EGFR |
| DH1 | PLA2G10 | DH2 | PDE4D | DH3 | HCK | DH5 | TGFBR2 |
| DH1 | EPHB4 | DH2 | CTSV | DH3 | GBA | DH5 | PPIA |
| DH1 | CHEK1 | DH2 | CA1 | DH3 | TREM1 | DH5 | BCHE |
| DH1 | PGR | DH2 | PLA2G10 | DH3 | AMY1A | DH5 | CTSV |
| DH1 | GBA | DH2 | GSR | DH3 | AMY1B | DH5 | CA12 |
| DH1 | CA12 | DH2 | PDE4B | DH3 | AMY1C | DH5 | GBA |
| DH1 | ESR2 | DH2 | HSP90AA1 | DH4 | GBA | DH5 | AMY1A |
| DH1 | KDR | DH2 | FAP | DH4 | CA12 | DH5 | AMY1B |
| DH1 | MTAP | DH2 | MMP13 | DH4 | NPR3 | DH5 | AMY1C |
| DH1 | PDE4B | DH2 | ESR2 | DH4 | FAP | DH5 | MTAP |
| DH1 | FKBP1A | DH2 | AMY1A | DH4 | GSTP1 | DH5 | NR1H2 |
| DH1 | DHFR | DH2 | AMY1B | DH4 | TREM1 | DH5 | CA1 |
| DH1 | ESRRG | DH2 | AMY1C | DH4 | PDE4B | DH5 | PDE4B |
| DH1 | IMPA1 | DH2 | BCHE | DH4 | MTAP | DH5 | HSP90AA1 |
| DH1 | ICAM2 | DH2 | AR | DH4 | NR1H2 | DH5 | PIM1 |
| DH2 | PPIA | DH2 | TGFBR2 | DH4 | CTSD |  |  |
| DH2 | CA2 | DH2 | FGFR1 | DH4 | ICAM2 |  |  |

Note: DH1 represents Catalpol D; DH2 represents Rehmannioside A; DH3 represents Rehmannioside; DH4 represents Melittoside; DH5 represents Ajugol
